# Novel Ultrafine-Grain Mg-Gd/Nd-Y-Ca Alloys with an Increased Ignition Temperature

**DOI:** 10.3390/ma16031299

**Published:** 2023-02-03

**Authors:** Stanislav Šašek, Peter Minárik, Jitka Stráská, Klára Hosová, Jozef Veselý, Jiří Kubásek, Robert Král, Tomáš Krajňák, Dalibor Vojtěch

**Affiliations:** 1 Department of Physics of Materials, Charles University, Ke Karlovu 5, 121 16 Prague, Czech Republic; 2 Research Centre, University of Žilina, Univerzitná 8215/1, 01026 Žilina, Slovakia; 3 Department of Metals and Corrosion Engineering, University of Chemistry and Technology, Technická 5, 166 28 Prague, Czech Republic

**Keywords:** magnesium, microstructure, ultrafine-grain, mechanical properties, flammability resistance

## Abstract

Two novel ignition-resistant magnesium alloys, Mg-2Gd-2Y-1Ca and Mg-2Nd-1Y-1Ca, were prepared in the ultrafine-grain condition by equal channel angular pressing (ECAP). In addition, four commercial alloys—AZ31, AX41, AE42 and WE43—were prepared similarly as a reference. The microstructure, mechanical properties and ignition temperature were thoroughly investigated. Both novel alloys exhibited a mean grain size of ~1 µm and dense distribution of small secondary phase particles. The mechanical strength measured by the tensile deformation test showed that the novel alloys are much stronger (~290 MPa) than all commercial alloys except WE43. However, Ca segregation into the grain boundaries caused a significant decrease in ductility (<6%). The ignition temperature of the novel alloys (~950 °C) was considerably improved by the presence of Gd/Nd, Y and Ca. This study showed that both novel alloys exhibit high strength and high ignition temperature in the ultrafine-grain condition.

## 1. Introduction

Magnesium and its alloys have recently been the subject of intensive research, primarily due to its low density ($\rho =1740\;\mathrm{kg}\cdot m^{-3}$) and associated high specific strength [1]. These properties make magnesium alloys an ideal candidate for industrial use emphasising high strength-to-weight ratio, e.g. the automotive or aerospace industries [2]. Using magnesium alloys can significantly reduce weight, resulting in lower costs and CO_2_ emissions reduction. The use of these materials in industrial applications could be even wider when the mechanical properties are enhanced by some microstructure refinement process, typically the severe plastic deformation (SPD) technique. The equal channel angular pressing (ECAP) is one of the most commonly used SPD techniques today and was proven to be efficient for magnesium alloys in preparation of ultrafine-grained microstructure [3,4].

However, the broader use of most commercial magnesium alloys in the automotive or aerospace industry is limited, particularly because of low corrosion- and flammability- resistance [2,5]. Until 2015, using magnesium alloys as a structural element in aircraft interiors was prohibited by Standard SAE Aerospace Standard (AS) 8049 C, but this regulation was revised. According to the new version, magnesium alloys may be used in aircraft seat construction if they meet the flammability performance requirements specified in DOT/FAA/AR-00/12.

It was found that some commercial alloys meet the abovementioned flammability requirements. The highest ignition resistance was measured for the WE43 alloy, which has also undergone extensive flammability testing by the FAA. Nevertheless, the known ignition-resistant alloys contain a high concentration of rare-earth metals (RE), significantly increasing their price. Therefore, there is a growing need for the development of novel ignition-resistant magnesium alloys with lower prices, that can be used in the automotive industry on a larger scale. There are three crucial parameters for the design of new magnesium alloys for the civil aircraft industry:

1. High flammability resistance,
2. Solid mechanical properties,
3. Acceptable price.

Despite the relatively intensive research in this area, no designed alloy has sufficiently fulfilled all three conditions. When magnesium is exposed to elevated temperatures, oxidation occurs. Oxidation is an exothermic reaction; therefore, heat is released during the process and thus the local temperature increases. This results in further acceleration of oxidation. If the oxidation rate is high enough that the surrounding material is unable to get rid of the released energy, a sharp increase in temperature occurs [5]. This phenomenon is considered an ignition. In addition to energy, another product of oxidation is an oxide layer. Magnesium oxide forms on the sample surface and prevents oxygen contact with magnesium atoms. However, magnesium oxide loses its structural integrity at about 600 °C [6]. It was found that the ignition resistance can be significantly increased by improving the oxide layer with the presence of a suitable oxide of different elements. The oxide layer of element A at the expense of MgO forms, if:

1. The formation of A_x_O_y_ oxide is favourable over MgO in terms of Gibbs free energy.
2. The solubility limit and diffusivity of alloying element A are sufficiently high.

Elements that met these criteria and are proven to be responsible for increasing flammability resistance are mainly rare-earth metals (RE) such as Y, Gd, Nd, Er [7,8,9,10]. Adding these elements also improves the mechanical properties [9,11]. On the other hand, because of their high price, it is advisable to use only a limited concentration of these elements.

A suitable alternative to RE is Ca. Ca is significantly cheaper than RE plus increases the strength and flammability resistance of magnesium alloys [12,13,14]. On the other hand, Ca tends to segregate on the grain boundaries, resulting in boundary-cohesion reduction, which may lead to alloy embrittlement [15]. Furthermore, common commercial alloys often contain elements that have detrimental effects on the flammability resistance. The most commonly-used element in this regard is Al. The decrease in the ignition resistance stems from the low melting temperature of the Mg-Al secondary-phase particles and the formation of melt capsules. The ignition mechanism is described in detail in [2].

The presented study aimed to develop novel ignition-resistant magnesium alloys with higher strengths and lower prices than the WE43 alloy. For this reason, two novel magnesium alloys, Mg-2Gd-2Y-1Ca and Mg-2Nd-1Y-1Ca, were prepared. Their composition was selected based on the literature review and previous results. Both alloys were prepared in the ultrafine-grain condition by ECAP processing. The selection of both Gd and Nd should have determined whether there is any difference in their effect on ignition temperature, and whether there is a different effect on the microstructural changes during the ECAP processing.

Note that the ignition temperature is not an intrinsic parameter of the given composition but depends on many factors, for example, the shape and size of the sample, temperature rate, atmospheric composition, and others [2,16,17]. For that reason, it is important to compare ignition temperatures measured with the same experimental setup. Therefore, this study comprised four commercial alloys with well-known properties: AZ31, AX41, AE42, and WE43. AZ31 is the most common magnesium alloy used in industry, AX41 and AE42 alloys were developed for high-temperature applications, and as mentioned above, the WE43 alloy passed the FAA ignition tests.

## 2. Materials and Methods

### 2.1. Material

Two novel magnesium alloys—Mg-2Gd-2Y-1Ca (VWX221) and Mg-2Nd-1Y-1Ca (NWX221)—along with the four commercial alloys—AZ31, AX41, AE42, WE43—were used in this study. The experimental alloys were cast from pure elements and master alloys (Mg-20Nd, Mg-30Y) by melting under an argon atmosphere. The commercial alloys were supplied in an extruded condition (AZ31, AX41, and AE42) or cast (WE43). The exact composition of all alloys was measured by energy-dispersive X-ray spectroscopy and is shown in Table 1.

All the investigated alloys were processed using equal channel angular pressing (ECAP). Bars with a square cross-section of 1 × 1 cm and length of 10 cm were processed, following route B_C_ (rotation of the sample by 90° between the individual passes), up to eight times to maximise the imposed strain. The die had inner and outer angles of 90° and 0°, respectively. The processing temperature and processing rate were tailored for each alloy separately. The novel alloys (VWX221, NWX221) were processed using similar parameters: the processing temperature was gradually decreased (from 340 °C to 290 °C), and the processing rate increased (from 2 mm min^−1^ to 7 mm min^−1^) after each processing step. The processing parameters of the commercial alloys were specified in our previous studies: AZ31 [18], AX41 [19], AE42 [20], and WE43 [11]. The ECAP die was equipped with an ejector that allows pushing the sample out of the die immediately after the processing. Molybdenum disulphide grease was used as a lubricant.

### 2.2. Microstructure

The microstructure of all samples was investigated using scanning electron microscopy (SEM) and transmission electron microscopy (TEM), including energy-dispersive X-ray spectroscopy (EDS) and electron backscatter diffraction (EBSD). In this study, we used a ZEISS (Oberkochen, Germany) Auriga Compact SEM equipped with EDAX EDS detector and EDAX EBSD camera (Pleasanton, CA, USA), and a JEOL 200F (Tokyo, Japan) TEM equipped with Gatan EDS detector. For both SEM and TEM, rectangular samples were cut from the central part of the ECAP-processed billets, and the investigation was performed on the cross-sectional plane. The samples for SEM were mechanically ground and polished down to 1 µm diamond paste and subsequently ion-polished using a Leica (Wetzlar, Germany) EM RES102. The samples for TEM were reduced to 150 µm thickness, and discs of 3 mm in diameter were cut out. Finally, Gatan (Pleasanton, CA, USA) Dimpler and Gatan PIPS were used to finish the samples.

The EBSD analysis was performed on the 100 × 100 µm area with a step size of 100 nm. The only exception was the WE43 alloy, which had to be analysed in the transmission mode; the scan size was 10 × 10 µm with a step size of 25 nm. The data analysis was primarily performed using the OIM Analysis 8 software. The raw data was partially cleaned by one step of confidence index (CI) standardisation and one iteration of grain dilatation, and only points with CI > 0.1 were used for the analysis. The texture analysis was performed in the MTEX 5.5.0 toolbox [21] implemented in the MATLAB 2022a software.

### 2.3. Mechanical Properties

Mechanical properties were investigated using a tensile deformation test, performed along the ECAP processing direction. Tensile tests were performed using a screw-driven Instron 5882 machine at the initial strain rate of 10^−3^ s^−1^ at room temperature. Flat specimens for tensile tests were machined from ECAP billets, with the lengthwise axis parallel to the pressing direction. The active part of the tensile samples was 12 × 3 × 1 mm for the VWX221, NWX221, and WE43 alloys. The tensile tests of the other three alloys were performed in previous studies; see AZ31 [18], AX41 [22], and AE42 [20].

### 2.4. Ignition Temperature

**Figure 1:**
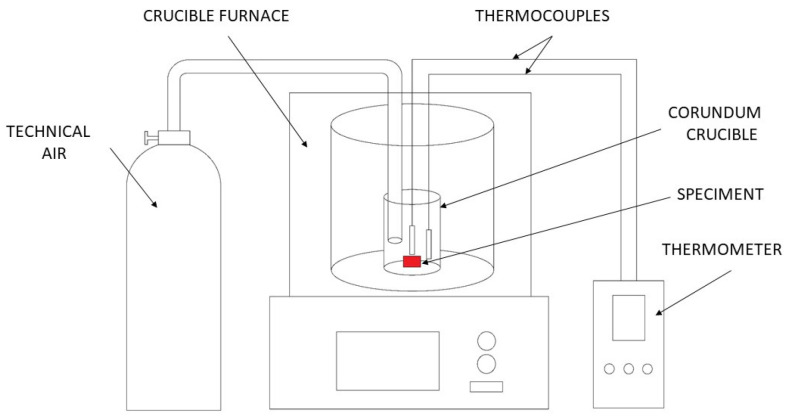
Schematic diagram of the ignition temperature measurement.

The ignition temperatures were measured by heating the samples in a resistance furnace with a constant rate of 8 K min^−1^. The samples were cylindrical, with a diameter of 5 mm and a height of 3 mm. The tested material was placed in an Al_2_O_3_ crucible, which was placed in the middle of the furnace, see Figure 1. During the test, technical air was supplied into the crucible at a flow rate of 100 l h^−1^. Two thermocouples (K-type) were placed near the surface of the sample. The sharp increase in temperature was interpreted to be the ignition temperature. At least three samples were measured from each alloy.

## 3. Results and Discussion

### 3.1. Microstructure

**Figure 2:**
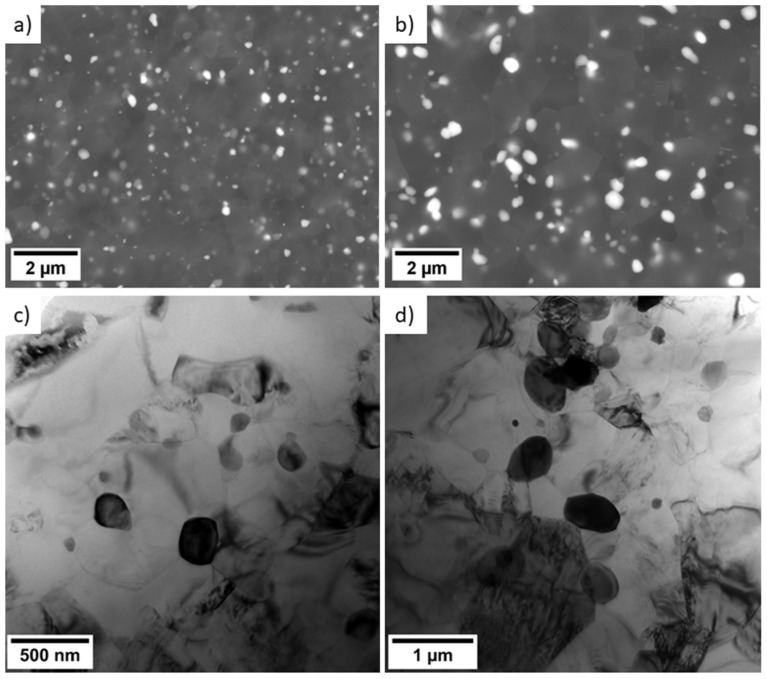
SEM (**a**,**b**) and TEM (**c**,**d**) images of the VWX221 (**a**,**c**) and NWX221 (**b**,**d**) alloys. Note different magnification in TEM images.

**Figure 3:**
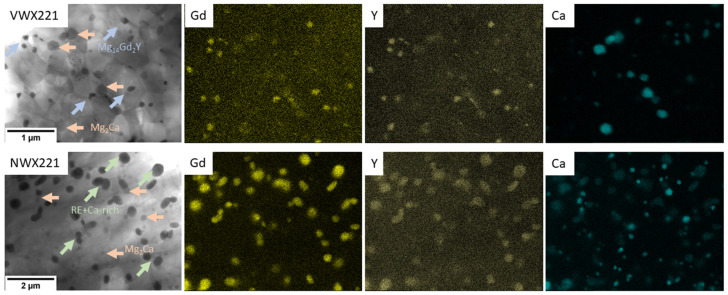
EDS elemental maps of the VWX221 and NWX221 alloys. Note different magnification.

The intense plastic deformation applied during the ECAP processing resulted in significant refinement of the microstructure with respect to both grain structure and secondary phase particles. Figure 2 shows the SEM and TEM micrographs of both novel alloys (VWX221, NWX221). Both samples show the dense distribution of small secondary-phase particles with oval shapes. The shape and distribution of the particles indicate that dissolution and reprecipitation of the secondary-phase particles occurred during the individual processing steps. A similar behaviour was repeatedly observed in magnesium alloys, which have a solvus temperature of the intermetallic phase not far above the processing temperature [11,23]. Regardless of the same processing parameters and comparable composition, it is evident that the Nd-containing alloy exhibits larger particles with a higher interparticle distance. Selected-area electron diffraction and local chemical analysis in TEM showed two types of intermetallic phases in the VWX221 alloy: Mg_2_Ca and Mg_14_Gd_2_Y. Figure 3 shows that both Y and Gd are also present in a relatively low concentration in Mg_2_Ca as well. The local chemical analysis revealed that their concentration is 0.2–0.5 at.%. At first glance, the secondary phase particles in the NWX221 alloy look like those in the VWX221 alloy. The material comprises the Mg_2_Ca phase, but the crystal structure of the RE-rich particles was not fcc (Mg_14_Nd_2_Y) but tetragonal. Figure 3 shows that Ca is present in the RE-rich particles, and the local chemical analysis of several particles showed that the ratio of Nd:Y:Ca is approximately 2:1:1. To the best of the Authors’ knowledge, this phase has not yet been recognised, and further work is needed to describe it fully.

**Figure 4:**
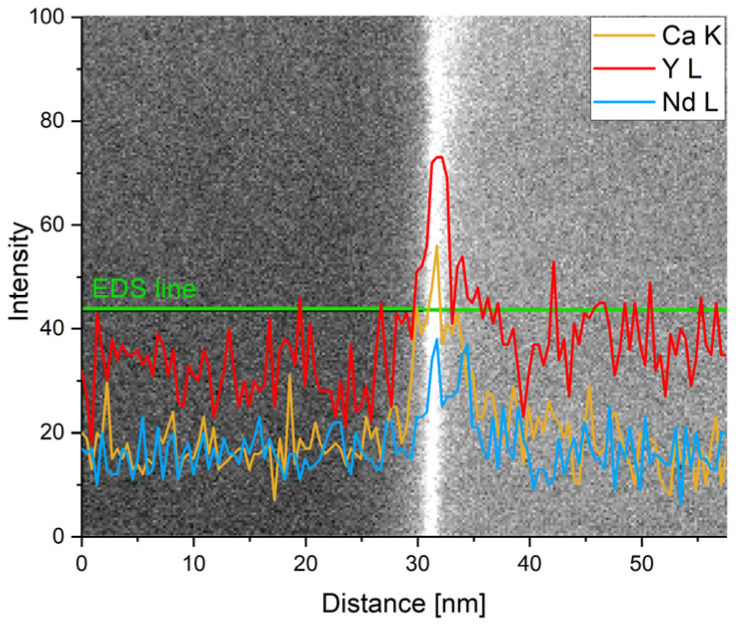
EDS elemental line profile across grain boundary, NWX221 sample.

The excessive amount of the alloying elements that dissolved into the magnesium matrix during processing tended to segregate into the grain boundaries. Increased content of all alloying elements (Gd/Nd, Y, and Ca) at the grain boundaries was observed in both alloys. EDS line scan across the grain boundary of the NWX221 sample is shown in Figure 4. Formation of this segregation is particularly important for mechanical properties, as discussed later.

**Figure 5:**
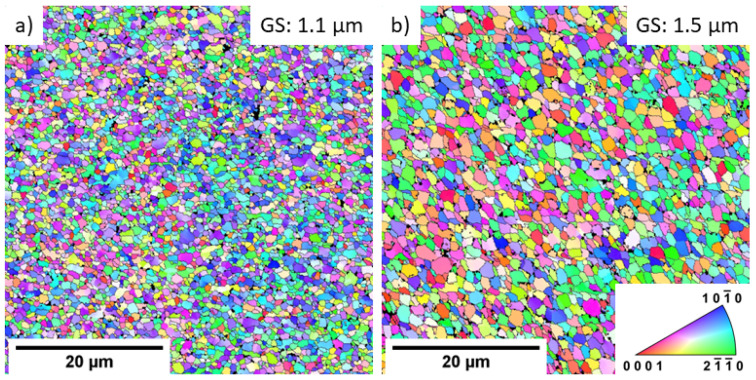
EBSD orientation maps of the (**a**) VWX221 and (**b**) NWX221 alloys.

The EBSD analysis of the grain structure showed that the ultrafine-grain condition was achieved in both alloys because of the ECAP processing. Figure 5 shows the corresponding EBSD orientation maps. Fully-recrystallised microstructure with equiaxed grains was observed in both alloys, and the calculated mean grain size was 1.1 µm and 1.5 µm for the VWX221 alloy and NWX221 alloy, respectively. The observed difference in the average grain size can be attributed to the difference in the size and distribution of the secondary phase particles: larger particles with higher interparticle distances were observed in the NWX221 alloy (Figure 2). The secondary-phase particles intensify grain nucleation during the processing and retard grain growth [24]. Therefore, the finer distribution of the particles observed in the VWX221 alloy was more effective for grain refinement. In addition, both materials exhibited distinct segregation of the alloying elements to the grain boundaries, which also strongly impacts the grain boundary movement and grain growth. Nevertheless, the magnitude of this effect was qualitatively comparable in both alloys; therefore, the difference in the development of the secondary phase particles’ distribution is considered the primary source of the difference in grain refinement.

**Figure 6:**
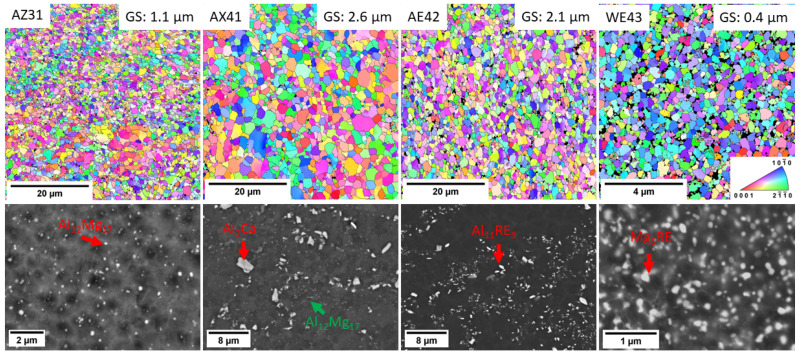
EBSD orientation maps and SEM images of AZ31, AX41, AE42 and WE43 commercial alloys processed by ECAP. Note different magnification of SEM images.

Figure 6 shows the EBSD orientation maps of the investigated commercial magnesium alloys processed by ECAP. The EBSD analysis showed that all studied samples exhibit uniform grain structure, which is homogeneous and is composed of small, nearly equiaxed grains. The mean grain sizes of the investigated alloys are in the range of 1.1–2.6 µm. The only exception is the alloy WE43 which has a mean grain size of 0.4 µm. Figure 6 also shows the distribution of the secondary phase particles for every material. Regardless of the composition, the secondary phase particles were significantly refined by the processing. It is essential to highlight that the particles in the AZ31 (Al_12_Mg_17_ phase) and WE43 (Mg_5_RE phase) alloys had an oval shape, similar to the novel alloys (VWX221, NWX221). On the other hand, the AE42 alloy exhibited fragmented particles with sharp edges. This difference is because the solvus temperature of the Al_11_RE_3_ phase is significantly higher than the processing one, and because this phase is not thermally affected during the processing, only mechanically. The relation between the processing and solvus temperature is seen in the AX41 alloy. The processing temperature was much lower than that of the VWX221 and NWX221 alloys, and the relatively large Al_2_Ca particles were more fragmented than dissolved. On the other hand, there is a very fine distribution of small Al_12_Mg_17_ particles between the large Mg_2_Ca ones because the solvus of the Al_12_Mg_17_ phase is much lower for this alloy composition [25]. The more thorough investigation focused on the interrelation between the ECAP parameters, phase structure and grain structure was already reported in previous papers: AZ31 [18], AX41 [19,26], AE42 [20], and WE43 [11].

### 3.2. Texture

**Figure 7:**
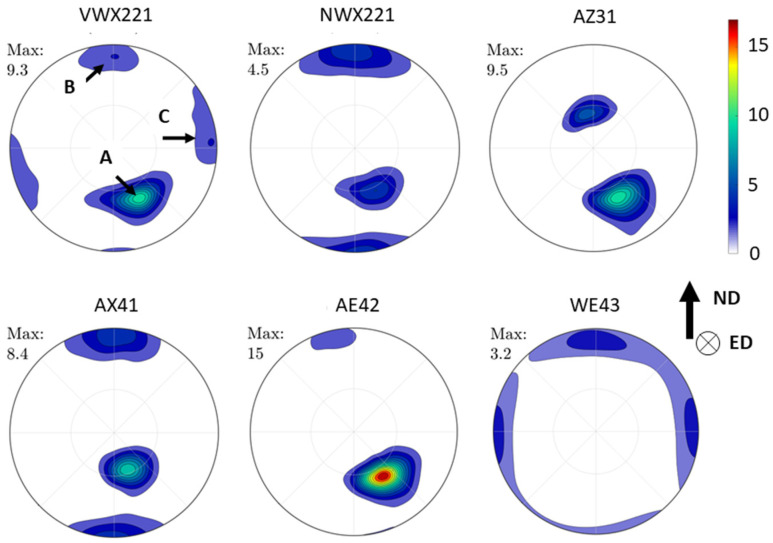
Pole figures of the investigated samples calculated from the EBSD data. The pole figure of the WE43 alloy was taken from our previous study [11] because of the too small EBSD data set.

Figure 7 shows pole figures for all investigated alloys. Three texture components were observed: denoted as A, B, and C. These texture components were repeatedly observed in magnesium alloys processed similarly. The texture component A was observed in all samples except the WE43 alloy. The strongest one was observed in the AE42 alloy (~15 times random), followed by AZ31 and VWX221 (~9.5 times random), and AX41 (~8.5 times random). The NWX221 alloy exhibited the lowest strength of this component (~4.5 times random). Components B and C were observed in alloys VWX221 (both), NWX221 (only B), AX41 (only B) and WE43 (both).

The texture component A is the most common in the ECAP-processed magnesium alloys, representing a (0001) fibre rotated approximately 50° from the processing direction and 40° from the vertical axis. This component forms by the predominant activation of basal slip during the processing [19]. The texture components B and C are formed because of the high activity of <a> prismatic and <c + a> second order pyramidal slip systems during the processing, respectively [20]. The formation of these components was observed primarily in magnesium alloys with rare-earth elements or lithium addition [20,24]. Nevertheless, the previous study shows that component B forms in the AX41 alloy because of a massive activity of the <c + a> second order pyramidal slip system during the processing [19]. The strong texture is, in general, not convenient. The highest strength was measured for the AE42 alloy, in which secondary-phase particles were large and were only mechanically fragmented by the processing. The results presented in this study are in line with the previous research [24], which shows that a decrease in texture strength may be associated with the dissolution and reprecipitation of small secondary phase particles, c.f. Figure 7, also Figure 2 and Figure 6. This knowledge is important for the future development of ultrafine-grain magnesium alloys with low crystallographic texture.

### 3.3. Mechanical Properties

**Figure 8:**
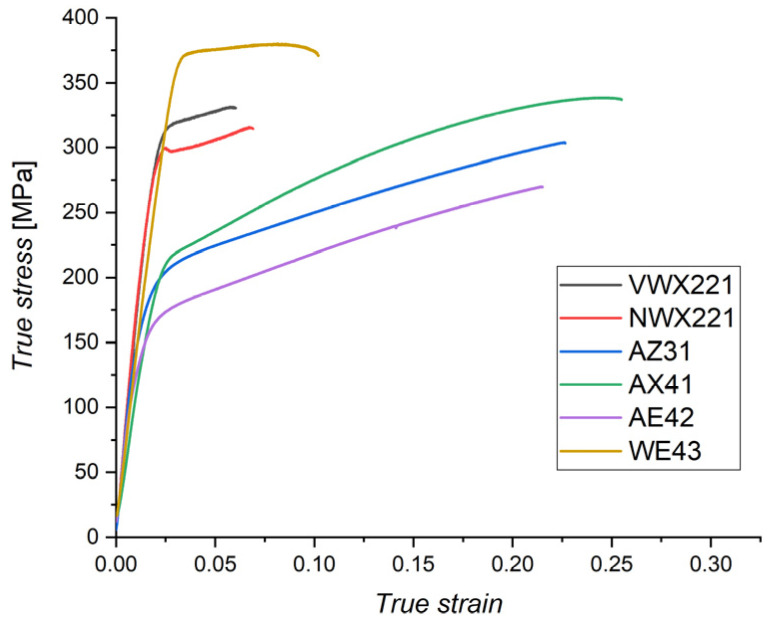
True stress–true strain curves of all investigated samples.

Mechanical properties of the studied alloys were investigated by uniaxial tensile tests along the ECAP processing direction. True stress—true strain curves for each alloy are shown in Figure 8. The tensile yield strength (TYS) was evaluated from the true stress—true strain curves and the ultimate tensile strength (UTS) was evaluated from the engineering deformation curves, see Table 2.

The novel alloys VWX221 and NWX221 showed very high TYS values: 303 ± 10 MPa and 282 ± 11 MPa, respectively. Their strength was much higher than that of the commercial AX41 (178 ± 8 MPa), AZ31 (168 ± 5 MPa), and AE42 (126 ± 9 MPa) alloys. The highest strength among the investigated alloys showed the commercial WE43 alloy: 356 ± 8 MPa. The strength of the samples was affected by the composition and especially by the microstructure. The grain boundary strengthening, following the Hall-Petch relation [27,28], is an essential factor for the strength of ultrafine-grain materials. The second very important parameter is the amount (distribution) of the secondary phase particles. At last, because ECAP processing often leads to the formation of strong texture, mechanical properties may be strongly affected by the texture, especially by component A during uniaxial tests.

The WE43 alloy showed the highest strength because of the combination of exceptionally small mean grain size (0.4 µm) and high density of very small secondary phase particles (Figure 6). The high strength of the novel alloys was due to the same effect of small grains and secondary phase particles, but the overall amount of the alloying elements was lower than in the case of the WE43 alloy. Therefore, the achieved mean grain size was higher, and the amount of secondary phase particles was lower. Consequently, a lower strength was observed in these two alloys than in the WE43 alloy. The VWX221 alloy showed higher strength than the NWX221 alloy, primarily because of smaller grain size and finer phase structure.

The effect of the secondary phase particles is also distinct in the case of the AX41 and AZ31 alloys. The AZ31 alloy had a much smaller mean grain size (Figure 6), but the yield strength was comparable. Microstructure investigation (Figure 6) showed that AX41 comprises Mg_2_Ca phase particles and a much higher amount of small Al_12_Mg_17_ phase particles than the AZ31 alloy. Compared to zinc, calcium has a lower solubility and thus has a greater tendency to form the secondary phase particles [25]. In addition, the AX41 alloy had a much higher amount of aluminium than the AZ31 alloy (Table 1), resulting in a higher amount of the Al_12_Mg_17_ phase particles. Considering comparable texture (Figure 7), these particles compensated for lower strengthening effect of the grain boundaries. Note that the precipitation hardening by Al_2_Ca particles has already been proved in previous reports [29,30,31]. The lowest strength measured for the AE42 alloy is because of the combined effect of lack of very small secondary phase particles, which were observed in other investigated alloys (Figure 2 and Figure 6), and the formation of very strong texture component A (Figure 7). This component was repeatedly found responsible for texture weakening during uniaxial straining along the processing direction [18,20].

The other important information resulting from Figure 8 is the deformability of the material. The alloys with superior strength (WE43, VWX221, NWX221) exhibited significantly lower ductility. This is because of the high amount of secondary-phase particles present predominantly along the grain boundaries. The particles cause strain localisation and facilitate crack initialisation and propagation. An even more pronounced decrease in the deformability measured for the VWX221 and NWX221 alloys (<6%) was caused by the segregation of the alloying elements into the grain boundaries, as shown in Figure 4. It was already reported that when calcium segregates into the grain boundaries, it causes a reduction in their cohesion [15]. The claim about the detrimental effect of calcium is also supported by the fact that Ca-free WE43 achieved significantly higher ductility, regardless of a much higher amount of the secondary phase particles.

### 3.4. Ignition Temperature

**Figure 9:**
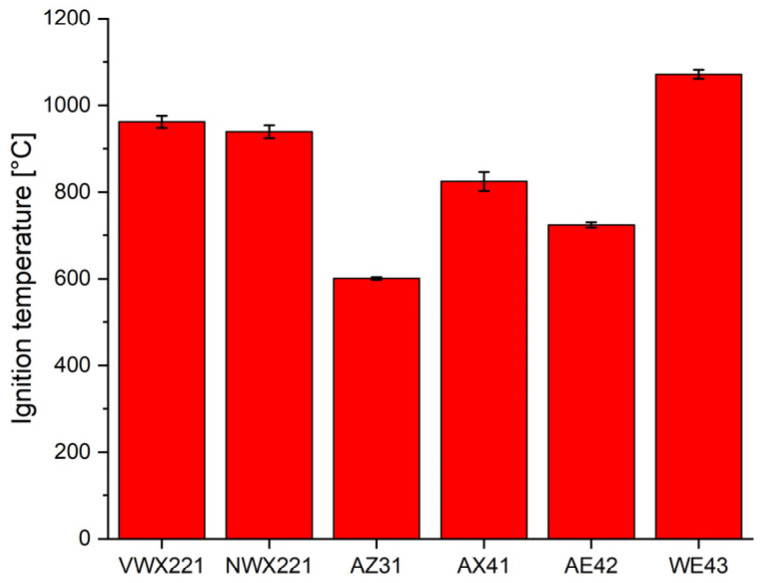
Ignition temperatures of the investigated alloys.

The investigated novel alloys VWX221 and NWX221 were designed to have a high ignition temperature, and for this reason, their ignition temperature was determined. As mentioned in the Introduction, the ignition temperature is not an intrinsic parameter of the given composition; therefore, for better comparison, this study comprises commercial alloys with known properties. The ignition temperatures measured by the linear heating test are shown in Figure 9.

The chemical composition of the alloy principally influences the ignition temperature. The highest value of the ignition temperature was achieved in the case of the WE43 alloy (1072 ± 10 °C), followed by VWX221 (962 ± 14 °C) and NWX221 (939 ± 15 °C). The other three commercial alloys showed significantly lower ignition temperatures: AX41 (825 ± 22 °C), AE42 (724 ± 6 °C), and AZ31 (601 ± 3 °C). The huge increase in the ignition temperature of the WE43, VWX221, and NWX221 alloys may be attributed to the formation of more stable oxides with protective character. MgO, the typical oxide forming on the surface of magnetism alloy exposed to high temperature, loses its structural integrity already below 600 °C [6]. On the other hand, it was repeatedly shown that the formation of Y_2_O_3_ [32,33], Gd_2_O_3_ and Nd_2_O_3_ [7,8,33], and CaO [16,34] might significantly increase the protective function of the oxide scale.

The high ignition temperature of the novel alloys was therefore achieved by the combined positive effect of the Gd/Nd, Y and Ca on the protectiveness of the formed oxide layer. The WE43 alloy exhibited even higher ignition temperature because of the higher amount of Y and RE elements than the VWX221 and NWX221 alloys. For the same reason, the AX41 and AE42 showed a lower ignition temperature. The lowest ignition temperature was measured for the AZ31 alloy because both aluminium and zinc do not contribute to the protective ability of the oxide scale forming on this alloy [2].

However, the effectiveness of the alloying elements with which they positively affect the oxide layer strongly depends on their availability during the oxidation process. The higher ignition temperature of the AX41 alloy than the AE42 alloy may be attributed to the difference in the dissolution of the Al_2_Ca phase and Al_11_RE_3_ phase. In both alloys, the alloying elements that enhance the oxide layer are at room temperature, bound to the secondary phase particles. However, in the case of the AX41 alloy, exceeding 517 °C results in the dissolution of Al_2_Ca and the release of Ca into the liquid phase [25]. In addition, approx. 1.1 wt.% of Ca is dissolved in the Mg matrix [25]. On the other hand, in the case of the AE42 alloy, the temperature at which the Al_11_RE_3_ phase starts to dissolve is 640 °C, and the solidus of the corresponding part of the Al-RE phase diagram is much steeper [25]. Consequently, RE elements are bound to the Al_11_RE_3_ phase for a longer time and cannot contribute to the oxide-scale protectiveness during the linear heating test as effectively as Ca in the AX41 alloy. According to the related phase diagrams [25], both AX41 and AE42 alloys are entirely melted at 650 °C. However, the linear heating test is a dynamic process, and the dissolution kinetics of the individual phases may significantly affect the resulting ignition temperature. Therefore, it is important to emphasise that secondary phase particles present in the novel alloys easily dissolve into the magnesium matrix and effectively protect the material from rapid oxidation and ignition.

## 4. Conclusions

Two novel ignition-resistant magnesium alloys, Mg-2Gd-2Y-1Ca and Mg-2Nd-1Y-1Ca, were prepared in the ultrafine-grain condition by equal channel angular pressing (ECAP) alongside four commercial alloys (AZ31, AX41, AE42, and WE43) which were used as a reference. The following conclusion may be drawn from this study:

- The grain structure of both alloys was refined by ECAP down to 1.1 µm and 1.5 µm for Mg-2Gd-2Y-1Ca and Mg-2Nd-1Y-1Ca, respectively. Both alloys contained dense distribution of small secondary phase particles. Higher refinement of Gd-containing alloy was attributed to its ability to form finer secondary phase particles.
- The mechanical strength of both novel alloys was very high: tensile-yield strength was ~290 MPa. However, the segregation of Ca into the grain boundaries caused a decrease in ductility below 6%. The strength of the novel alloys was much higher than the commercial ones, except for the WE43, but the ductility was lower.
- The ignition temperature of both novel alloys (~950 °C) was significantly higher than that of the commercial alloys, thanks to the combined effect of Gd/Nd, Y and Ca. The only exception was the WE43 alloy, which showed a higher ignition temperature because of the much higher amount of rare-earth elements and yttrium.
- This study showed that both alloys could be prepared in the condition exhibiting high strength and high ignition temperature. It was found that there is a small difference in the effect of Gd and Nd on the microstructure during the ECAP processing, but the strength and ignition temperature of both alloys was similar.

## Figures and Tables

**Table 1:** Chemical composition of all investigated alloys measured by energy-dispersive X-ray spectroscopy in wt%.

	Y	Gd	Nd	Ce	Ca	Al	Zn	Zr	Mg
VWX221	1.8	2.2	-	-	0.9	-	-	-	Bal.
NWX221	1.5	-	2.0	-	1.0	-	-	-	Bal.
AZ31	-	-	-	-	-	2.8	0.9	-	Bal.
AX41	-	-	-	-	0.9	5.6	-	-	Bal.
AE42	-	-	0.6	1.3	-	3.5	-	-	Bal.
WE43	3.8	0.6	2.3	-	-	-	-	0.4	Bal.

**Table 2:** Mechanical properties calculated from the tensile curves. TYS was evaluated from the true stress–true strain curves and UTS from the engineering deformation curves.

	TYS [MPa]	UTS [MPa]
VWX221	303 ± 10	308 ± 10
NWX221	282 ± 11	284 ± 12
AZ31	168 ± 5	245 ± 6
AX41	178 ± 8	270 ± 26
AE42	126 ± 9	220 ± 8
WE43	356 ± 8	367 ± 14

## Data Availability

The data presented in this study are available at meaningful request from the corresponding author.
